# Efficacy of melflufen in multiple myeloma with mutated or deleted *TP53*

**DOI:** 10.1186/s40164-025-00729-1

**Published:** 2025-12-23

**Authors:** Klara Acs, Juho J. Miettinen, Philipp Sergeev, Tobias Heckel, Yumei Diao, Kristina Witt-Mulder, Marcus Thureson, Thorsten Bischler, Maiju-Emilia Huppunen, Minna Suvela, Jakob Obermüller, Umair Munawar, Ana Slipicevic, Ralf C. Bargou, Fredrik Lehmann, Stefan Svensson Gelius, Stefan Norin, Fredrik Schjesvold, Pieter Sonneveld, Thorsten Stühmer, Caroline A. Heckman

**Affiliations:** 1 Oncopeptides AB, Stockholm, Sweden; 2 Institute for Molecular Medicine Finland, Helsinki Institute of Life Science, iCAN Digital Precision Cancer Medicine Flagship, University of Helsinki, Helsinki, Finland; 3 Core Unit Systems Medicine, University of Würzburg, Würzburg, Germany; 4 Comprehensive Cancer Center Mainfranken, Würzburg, Germany; 5 Oslo Myeloma Center, Department of Hematology, Oslo University Hospital, Oslo, Norway; 6 Department of Hematology, Erasmus MC Cancer Institute, Rotterdam, The Netherlands; 7 Present Address: ZalVac AB, Solna, Sweden; 8 Present Address: One-carbon Therapeutics AB, Stockholm, Sweden; 9 Present Address: Industrifonden, Stockholm, Sweden

**Keywords:** Melflufen, Clinical efficacy, *TP53* mutation, del(17p), PFS, In vitro efficacy, SoC alkylators

## Abstract

Patients with multiple myeloma bearing a deletion of chromosome 17p (del(17p)), mutation of *TP53*, or both have poorer prognosis compared to patients without these aberrations. We investigated the activity and mechanism of melflufen (melphalan flufenamide) in myeloma models with wild type *TP53* (*TP53*wt) and complete *TP53* deletion (*TP53*^−/−^) and assessed the efficacy of melflufen in patients with del(17p) and/or *TP53* mutation. Ex vivo data from myeloma plasma cells (PC) showed comparable activity of melflufen in del(17p), *TP53*^*−/−*^, and *TP53*wt samples. scRNAseq data demonstrated that melflufen sensitive PCs had lower expression of p53 target genes and higher expression of genes associated with DNA damage repair and cell cycle checkpoints. Irrespective of *TP53* status, melflufen induced apoptosis, DNA damage, and mitochondrial dysfunction, while only in *TP53*^−/−^ cells, it led to changes in expression of cell cycle checkpoint and apoptosis genes. Post-hoc analysis of the OCEAN trial melflufen-treated del(17p) patient population also demonstrated favorable progression free survival compared to pomalidomide-treated cohort. Our insights into the molecular mechanisms of melflufen activity in *TP53*^*−/−*^ myeloma support its clinical efficacy and application in the del(17p) and *TP53*^*−/−*^ patient population.

*Trial registration* NCT03151811, registration 2017-05-09.

**Supplementary Information:**

The online version contains supplementary material available at 10.1186/s40164-025-00729-1.

To the editor,

Deletion of chromosome 17p (del(17p)) in multiple myeloma is a well-known poor prognostic factor, primarily due to the loss of the *TP53* gene, which encodes the p53 tumor suppressor protein, critical for maintaining genomic integrity and regulating apoptosis [1]. Patients with del(17p) are classified as high-risk, and prognosis worsens significantly when a mutation occurs in *TP53* on the remaining allele (“double-hit”), resulting in complete loss of functional p53 [2–5].

The phase III OCEAN trial compared melflufen plus dexamethasone (mfldex) with pomalidomide plus dexamethasone (pomdex) in patients with relapsed/refractory multiple myeloma (RRMM), demonstrating superior progression-free survival (PFS) with melflufen [6, 7]. Post-hoc analyses further revealed improved PFS and overall survival (OS) in patients without prior autologous stem cell transplant (ASCT) or with disease progression occurring more than 36 months post-ASCT [8].

Given melflufen’s potent cellular efficacy, we investigated its mechanism of action in models with and without *TP53* mutation, including RRMM patient plasma cells (PCs) and isogenic cell lines with deletion of *TP53.* In addition, we analysed outcome data from patients with del(17p)/*TP53* aberrations who were enrolled in the OCEAN trial.

Ex vivo drug testing of bone marrow (BM) samples from patients with myeloma (Fig. S1A) showed that melflufen retained superior efficacy towards BM PCs across all samples, regardless of *TP53* mutation, del(17p) status, or other checked aberrations, outperforming melphalan and cyclophosphamide (Fig. 1A, S1B). Using single-cell RNA sequence data of the BM samples, gene set enrichment analysis (GSEA) of differentially expressed genes between high-sensitive (HS) and low-sensitive (LS) samples revealed downregulation of p53 downstream targets in the HS group and enrichment of DNA damage repair (DDR) pathways involving *BRCA1*, *ATM*, and *CHEK2* (Fig. 1B).

To evaluate the impact of *TP53* aberrations on cell response to melflufen, we employed the AMO-1 myeloma cell line with either *TP53*wt or *TP53*^-/-^ and compared baseline gene expression profiles between *TP53*wt or *TP53*^-/-^ patient samples and cell lines. Across both comparisons (Tables S1, S2), 252 genes were similarly deregulated, including *IL6R*, *JUN*, *DUSP5*, *AREG*, and *FOS* (Table S3). GSEA of these genes highlighted pathways related to *TP53*, cell cycle progression, signalling, and DNA damage (Figs. 1C, S2), confirming consistent effects of p53 dysfunction in both cell lines and patient samples.

Consistent with previous reports [9, 10], *TP53*^-/-^ AMO-1 cells exhibited profound resistance to melphalan (ΔEC50 ≥ 11 µM), but not to melflufen (ΔEC50 = 0.5 µM) (Figures S3A, S3B; Table S4). Early- (Fig. 2A) and late-stage (Fig. 2B) apoptosis was also induced by melflufen more effectively and at lower doses, compared to melphalan and cyclophosphamide, aligning with prior studies [11, 12]. Furthermore, melflufen triggered DNA damage signaling (H2AX phosphorylation (γH2AX) at Ser139) in *TP53*wt cells in a dose-dependent manner, whereas in *TP53*^-/-^ cells, maximal effect was observed even at the lowest concentration (Fig. 2C). These cells also exhibited elevated baseline γH2AX levels, consistent with p53 loss (Figure S3C), which explains the lower added value of treatment in this model [1]. Melphalan and cyclophosphamide induced less early DNA damage in both models.

Mitochondrial membrane potential measurements revealed that melflufen caused a rapid decline in both *TP53*wt and *TP53*^-/-^ cells within 2 h, with a more pronounced effect in *TP53*^-/-^ cells (Fig. 2D), signifying decline in mitochondrial functionality. In contrast, melphalan and cyclophosphamide had minimal impact.

Following 2-hour exposure to IC90 concentrations of melflufen or melphalan (Table S4), melflufen-treated cells exhibited distinct gene expression changes in p53 and DNA damage repair pathways, particularly in *TP53*^-/-^ cells (Figure S4), which persisted after 12 h. These patterns underscore a mechanistic divergence between the two alkylators.

Finally, to validate our preclinical findings, we performed a post-hoc analysis of the OCEAN trial. In the del(17p) subgroup, mfldex significantly improved PFS (7.1 vs. 2.9 months, HR 0.45 [95% CI: 0.26, 0.79], *p* = 0.006) and overall response rate (ORR: 33.3% vs. 10.8%, *p* = 0.028) compared to pomdex (Figures S5A, B), confirming its higher efficacy. Limiting del(17p) to ≥ 50% retained ORR and PFS significance despite reduced cohort size (Figure S6). In the cohort with co-occurrence of del(17p) and + 1q, separate analysis of + 1q patients showed no mfldex–pomdex differences, confirming del(17p) as the main driver (Figure S7). For information about the patient cohort see Tables S5-7.

In conclusion, our data highlight melflufen’s mechanism of action – intracellular accumulation of its alkylating payload leading to rapid DNA damage and mitochondrial dysfunction, independent of *TP53* status. These findings, supported by clinical data from the OCEAN trial, suggest that melflufen is particularly effective in high-risk myeloma with del(17p) and/or *TP53* mutation.

**Fig. 1:**
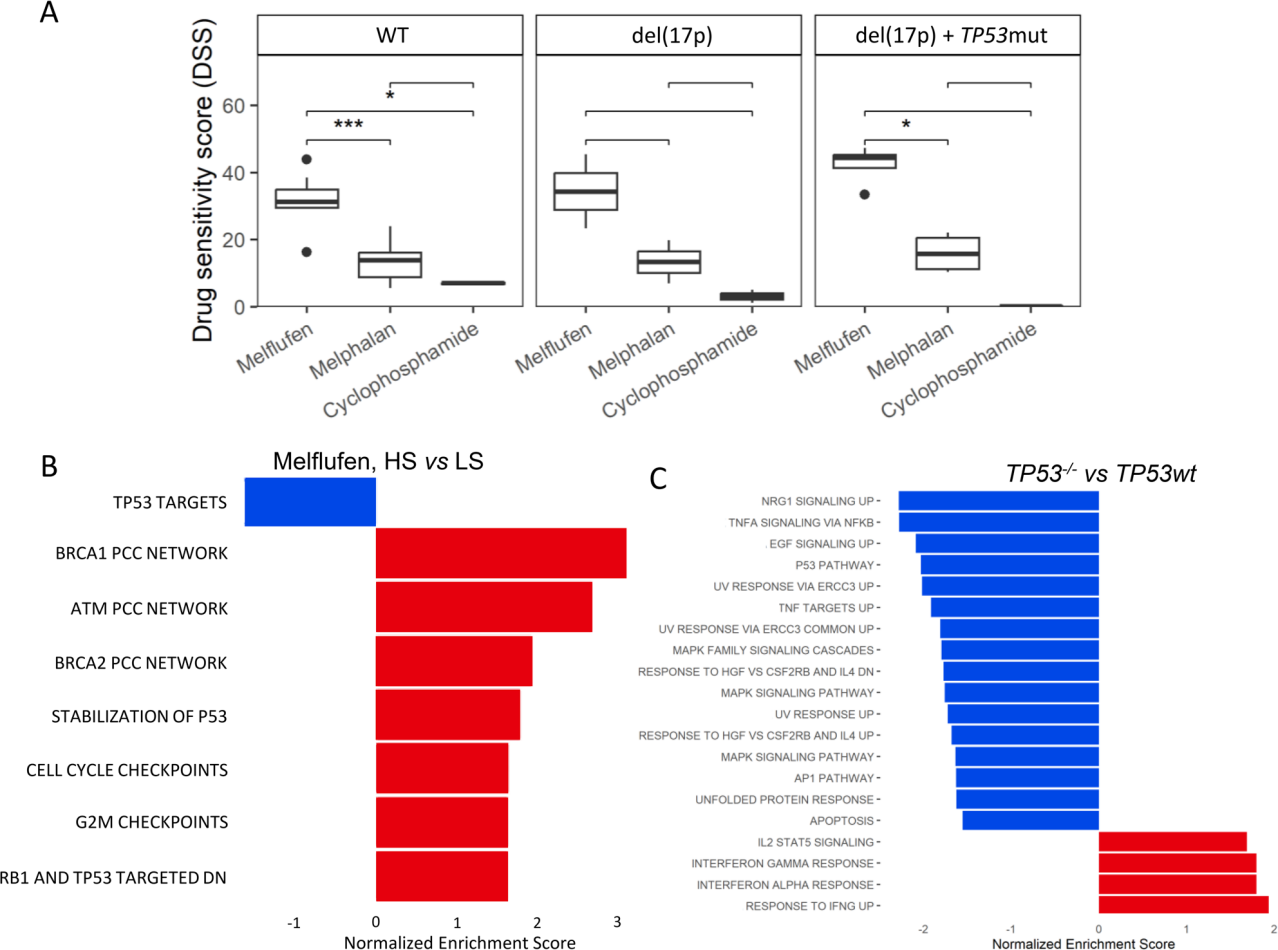
Differential sensitivity to alkylators and enriched gene sets in plasma cells from 24 myeloma bone marrow samples. **A)** Drug sensitivity scores of samples with different molecular background for three alkylators – melflufen, melphalan, and cyclophosphamide. P-values are indicated above the brackets, with the corresponding comparison. * – *p* < 0.05, *** – *p* < 0.001. **B)** Bar plot, representing normalized enrichment scores for selected gene sets, derived from the most differentially expressed genes in the PC populations between the melflufen highly sensitive (HS) and low sensitive (LS) groups. **C)** Bar plots, representing normalized enrichment score for top 20 deregulated gene sets, consisting of commonly deregulated genes in cell lines and patient samples *TP53*wt versus *TP53*^*−/−*^

**Fig. 2:**
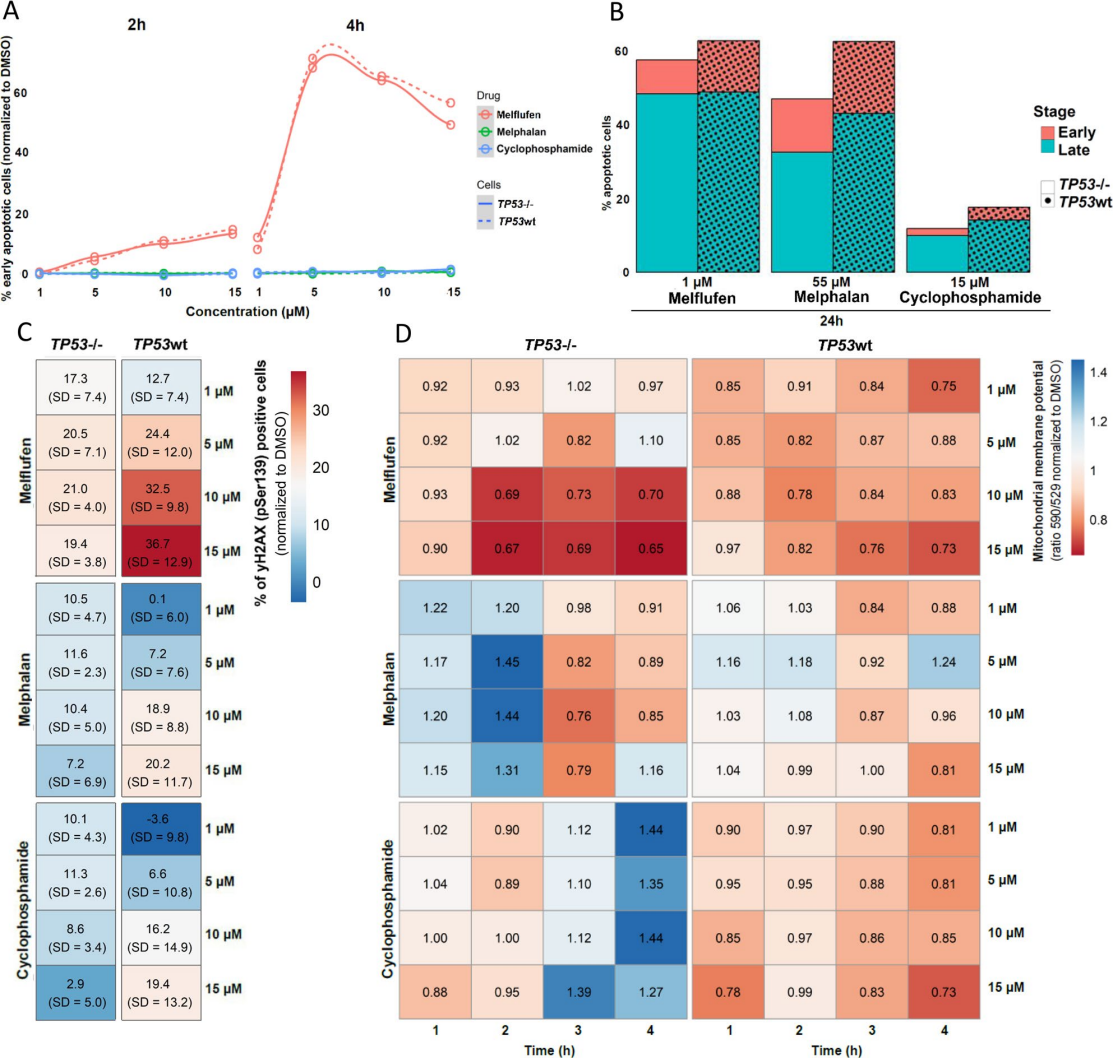
Melflufen-induced apoptosis, DNA damage. and mitochondrial dysfunction in *TP53* isogenic cell line models, **A)** Melflufen, melphalan or cyclophosphamide induced early apoptosis in AMO-1 *TP53*wt and AMO-1 *TP53*^−/−^ isogenic cell lines. Cells were treated with the indicated drug concentrations and time points, melflufen (red), melphalan (green), or cyclophosphamide (blue). Early apoptosis was detected with Annexin V and PI staining using flow cytometry (Annexin V positive, PI negative). **B)** Both early and late apoptosis were measured in AMO-1 *TP53*wt and AMO-1 *TP53*^−/−^ isogenic cell lines after 24 h treatment with the indicated drugs and drug concentrations (late apoptotic cells: Annexin V positive, PI positive). **C)** Melflufen, melphalan or cyclophosphamide induced DNA damage in AMO-1 *TP53*wt and AMO-1 *TP53*^−/−^ isogenic cell lines. Data was normalized to DMSO, and average results are presented from 0.5, 1, 2, and 4 h incubation time points. Early DNA damage was detected as yH2AX signal by flow cytometry after the indicated treatment concentrations and time points. yH2AX: phosphorylated histone protein 2AX. **D)** Melflufen, melphalan or cyclophosphamide treatment induced mitochondrial disfunction in AMO-1 *TP53* isogenic cell line. Melflufen, melphalan or cyclophosphamide treatment were applied in the indicated concentrations, and after 1, 2, 3 and 4 h time points mitochondrial function of the treated cells was measured using JC-1 mitochondrial membrane potential assay. PI: propidium iodide

## Supplementary Information

Below is the link to the electronic supplementary material.

Supplementary Material 1

Supplementary Material 2

Supplementary Material 3

Supplementary Material 4

Supplementary Material 5

Supplementary Material 6

Supplementary Material 7

Supplementary Material 8

Supplementary Material 9

Supplementary Material 10

Supplementary Material 11

Supplementary Material 12

Supplementary Material 13

Supplementary Material 14

Supplementary Material 15

## Data Availability

Bulk RNAseq data from AMO1 *TP53* wt cells and the **TP53*-/-* knock out clonal subline AMO-1 TP53-/- is available at the NCBI GEO (http://www.ncbi.nlm.nih.gov/geo) accession number GSE254959. Single cell RNA sequencing data from multiple myeloma patient samples is available at the NCBI GEO (http://www.ncbi.nlm.nih.gov/geo) accession number GSE263201. All other data are available upon request from the corresponding author.

## Abbreviations

del(17p): Deletion of the short arm of chromosome 17
*TP53*wt: Wild-type *TP53*
*TP53*^−/−^: *TP53* knockout (biallelic deletion)
PC: Plasma cells
scRNAseq: Single-cell RNA sequencing
mfldex: Melflufen plus dexamethasone treatment regimen
pomdex: Pomalidomide plus dexamethasone treatment regimen
RRMM: Relapsed/refractory multiple myeloma
PFS: Progression-free survival
OS: Overall survival
ASCT: Autologous stem cell transplant
GSEA: Gene set enrichment analysis
DDR: DNA damage repair
HS: High-sensitive samples
LS: Low-sensitive samples
IC90: Inhibitory concentration 90%
ORR: Overall response rate
HR: Hazard ratio
CI: Confidence interval
