# Supplementary material for: Efficacy of melflufen in multiple myeloma with mutated or deleted TP53
Source: Exp Hematol Oncol. 2025 Dec 23;14:138. doi: 10.1186/s40164-025-00729-1 (PMC12729255; doi:10.1186/s40164-025-00729-1)
Supplement: Supplementary file 1 — Supplementary Material 1 [file 40164_2025_729_MOESM1_ESM.pdf]

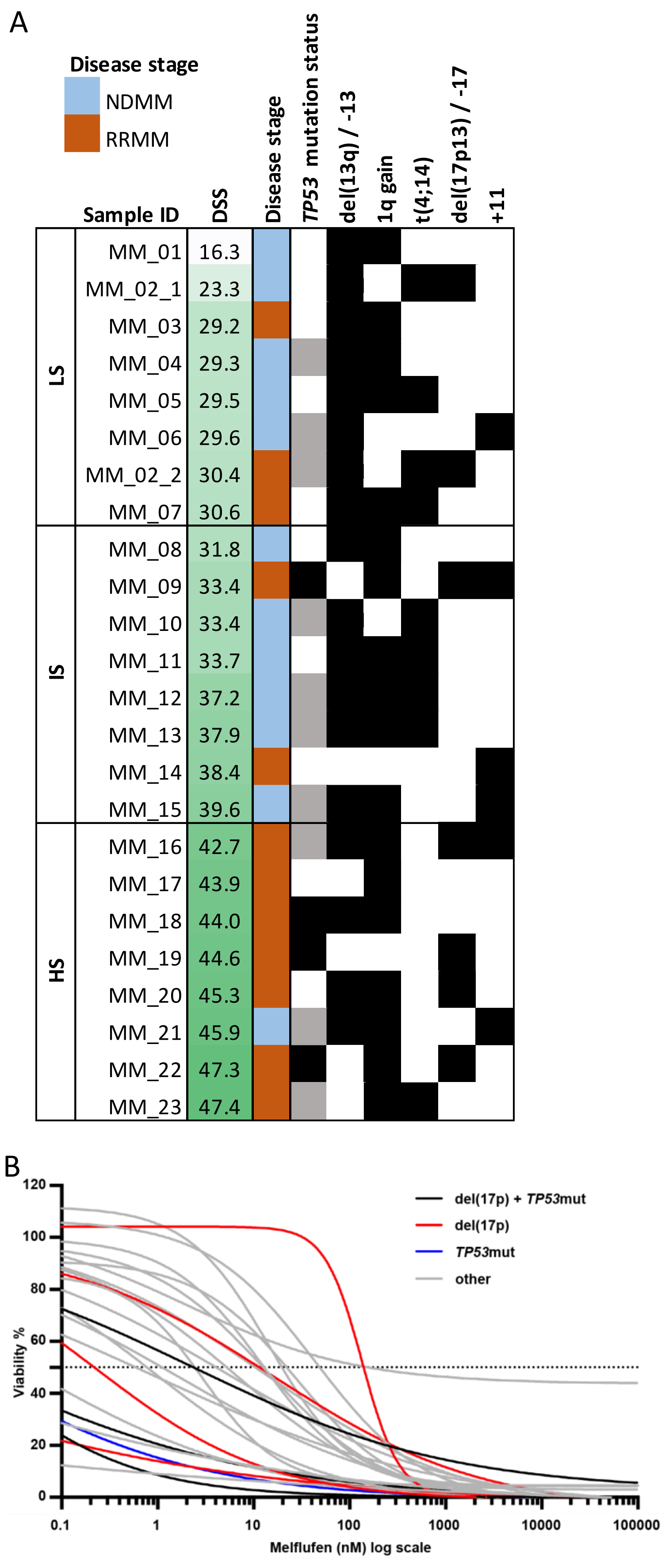
A
Disease stage
NDMM
RRMM
Sample ID
DSS
Disease stage
TP53 mutation status
del(13q) / -13
1q gain
t(4;14)
del(17p13) / -17
+11
LS
IS
HS
MM_01 16.3
MM_02_1 23.3
MM_03 29.2
MM_04 29.3
MM_05 29.5
MM_06 29.6
MM_02_2 30.4
MM_07 30.6
MM_08 31.8
MM_09 33.4
MM_10 33.4
MM_11 33.7
MM_12 37.2
MM_13 37.9
MM_14 38.4
MM_15 39.6
MM_16 42.7
MM_17 43.9
MM_18 44.0
MM_19 44.6
MM_20 45.3
MM_21 45.9
MM_22 47.3
MM_23 47.4
B
del(17p) + TP53mut
del(17p)
TP53mut
other
Viability %
120
100
80
60
40
20
0
0.1
1
10
100
1000
10000
100000
Melflufen (nM) log scale
