# Supplementary figures and images for: Efficacy of melflufen in multiple myeloma with mutated or deleted TP53
Source: Exp Hematol Oncol. 2025 Dec 23;14:138. doi: 10.1186/s40164-025-00729-1 (PMC12729255; doi:10.1186/s40164-025-00729-1)

A

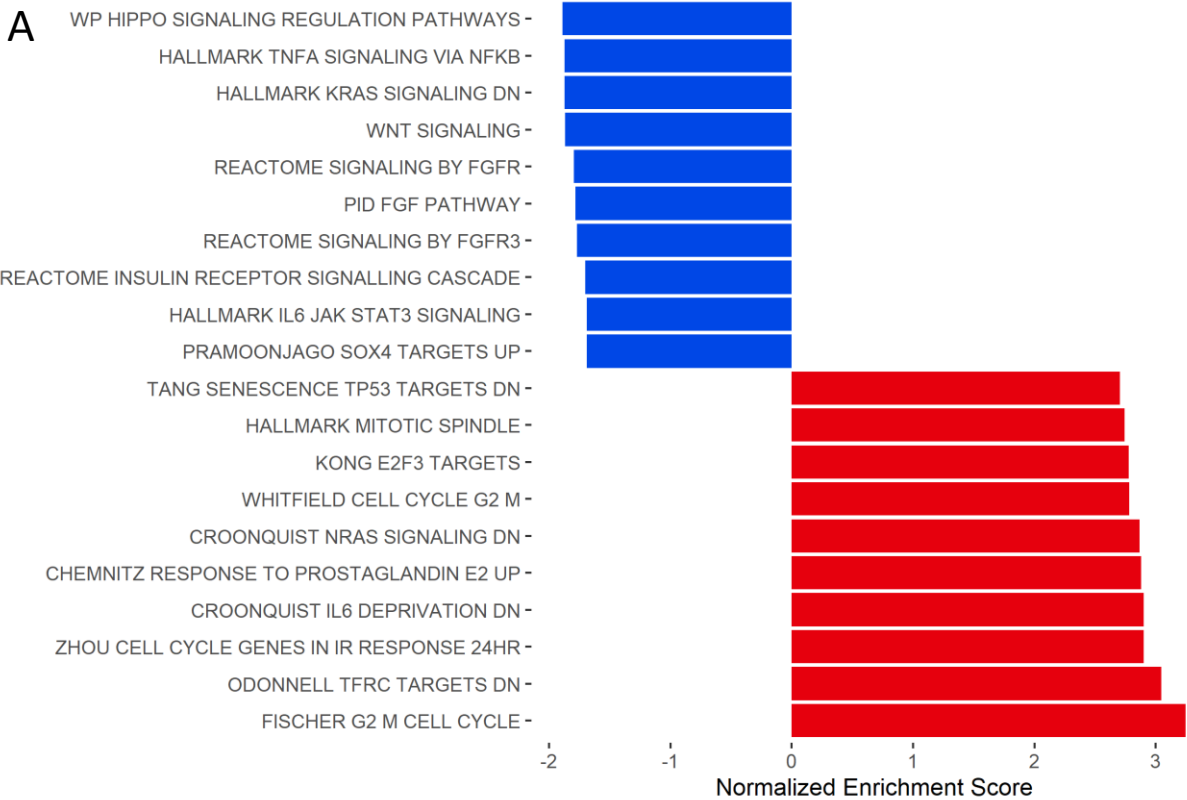

B

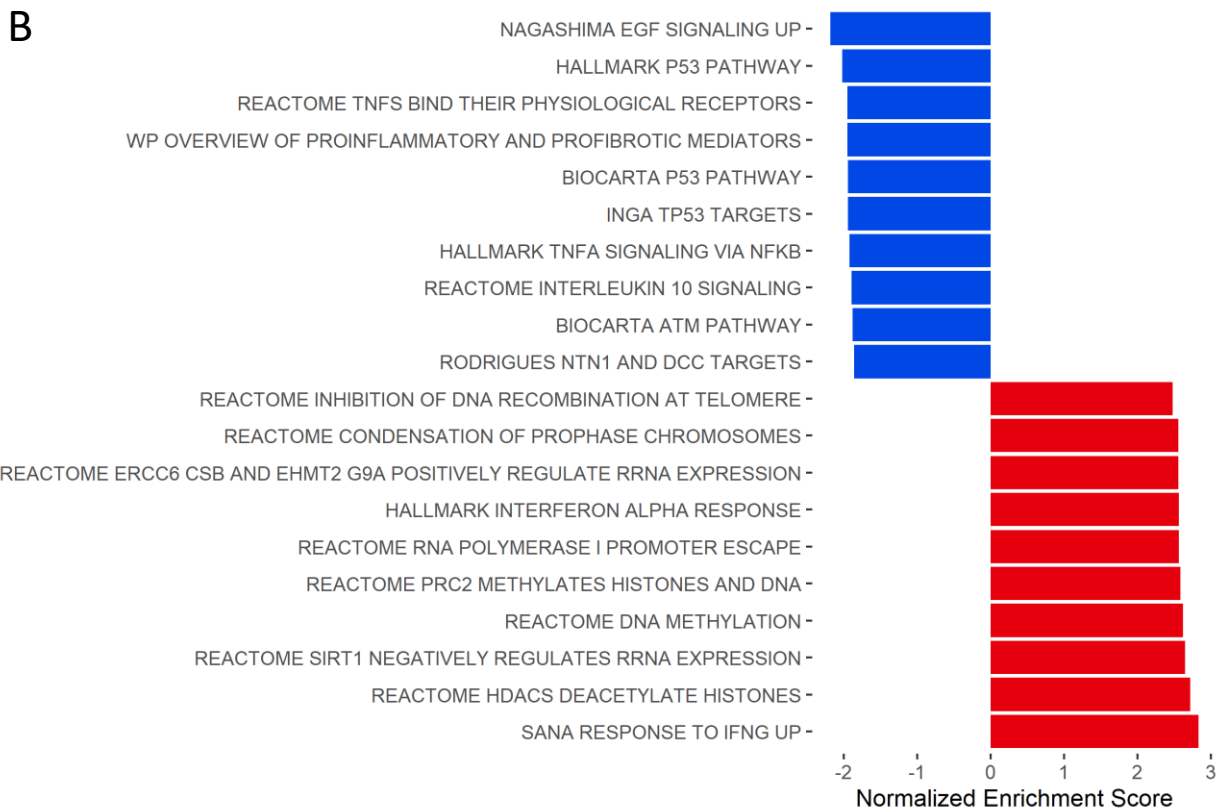

Supplement: Supplementary file 2 — Supplementary Material 2 [file 40164_2025_729_MOESM2_ESM.pdf]

**A**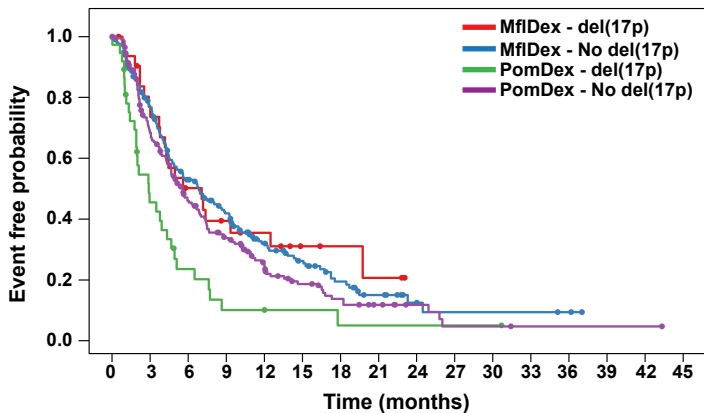

**Patients at risk**

|                      |     |     |    |    |    |    |    |    |   |   |   |   |   |   |   |   |
|----------------------|-----|-----|----|----|----|----|----|----|---|---|---|---|---|---|---|---|
| MflDex - del(17p)    | 33  | 23  | 14 | 10 | 8  | 4  | 3  | 2  | 0 | 0 | 0 | 0 | 0 | 0 | 0 | 0 |
| MflDex - No del(17p) | 213 | 145 | 95 | 70 | 42 | 30 | 19 | 11 | 5 | 3 | 3 | 3 | 2 | 0 | 0 | 0 |
| PomDex - del(17p)    | 37  | 15  | 7  | 3  | 3  | 2  | 1  | 1  | 1 | 1 | 1 | 0 | 0 | 0 | 0 | 0 |
| PomDex - No del(17p) | 212 | 135 | 83 | 55 | 34 | 21 | 14 | 9  | 5 | 2 | 2 | 1 | 1 | 1 | 1 | 0 |

**B**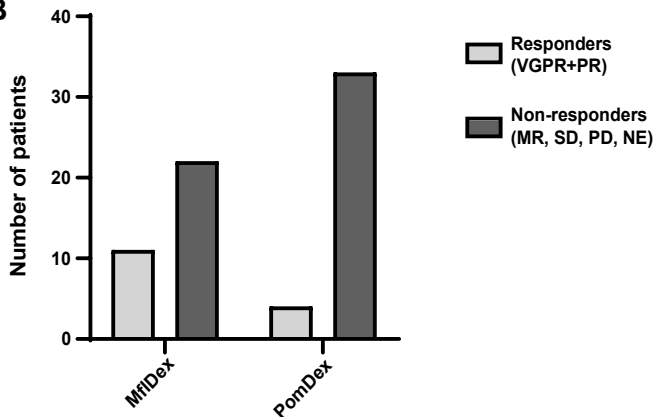

Supplement: Supplementary file 3 — Supplementary Material 3 [file 40164_2025_729_MOESM3_ESM.pdf]

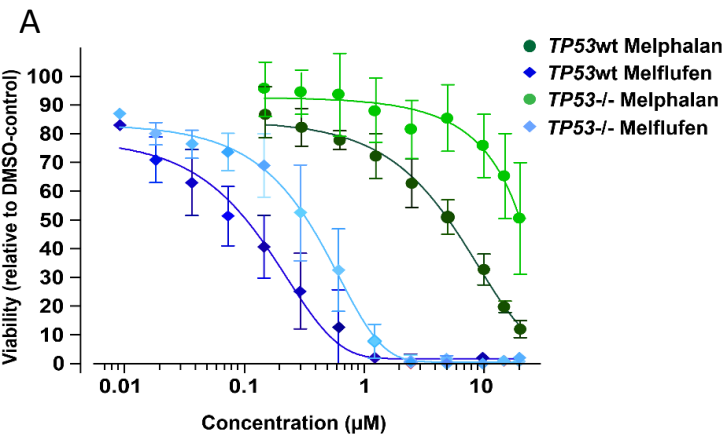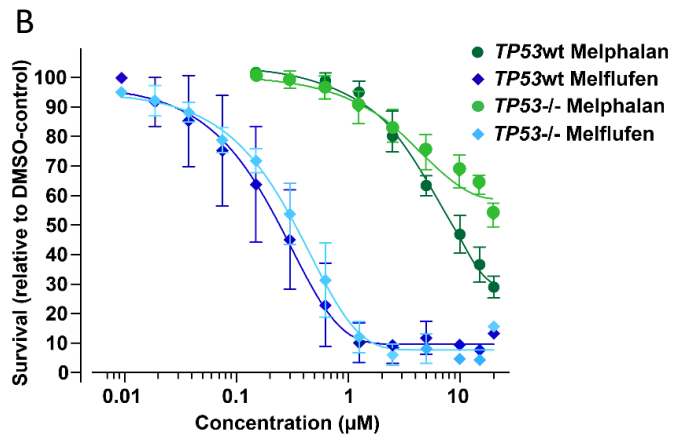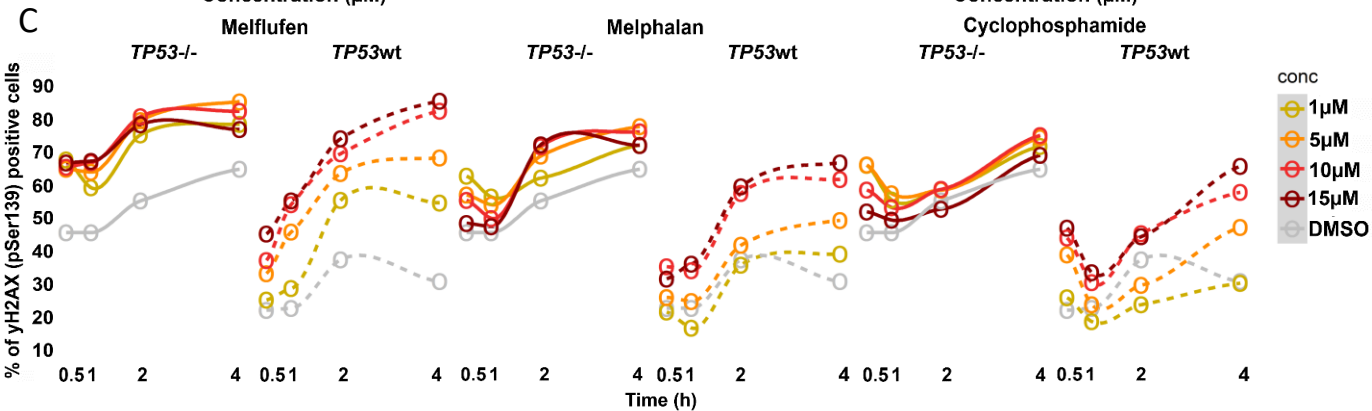

Supplement: Supplementary file 4 — Supplementary Material 4 [file 40164_2025_729_MOESM4_ESM.pdf]

p53 signalling pathway

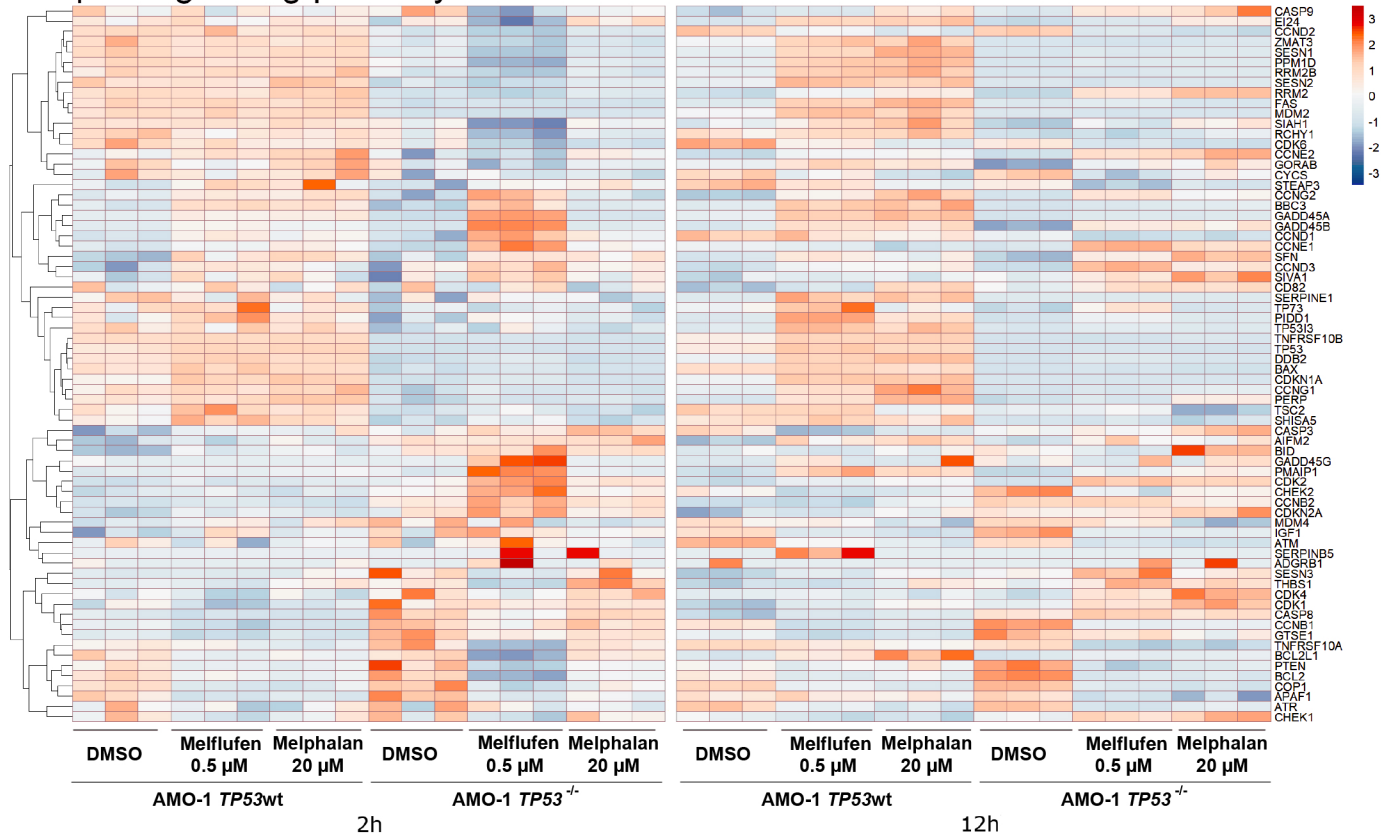

DNA replication

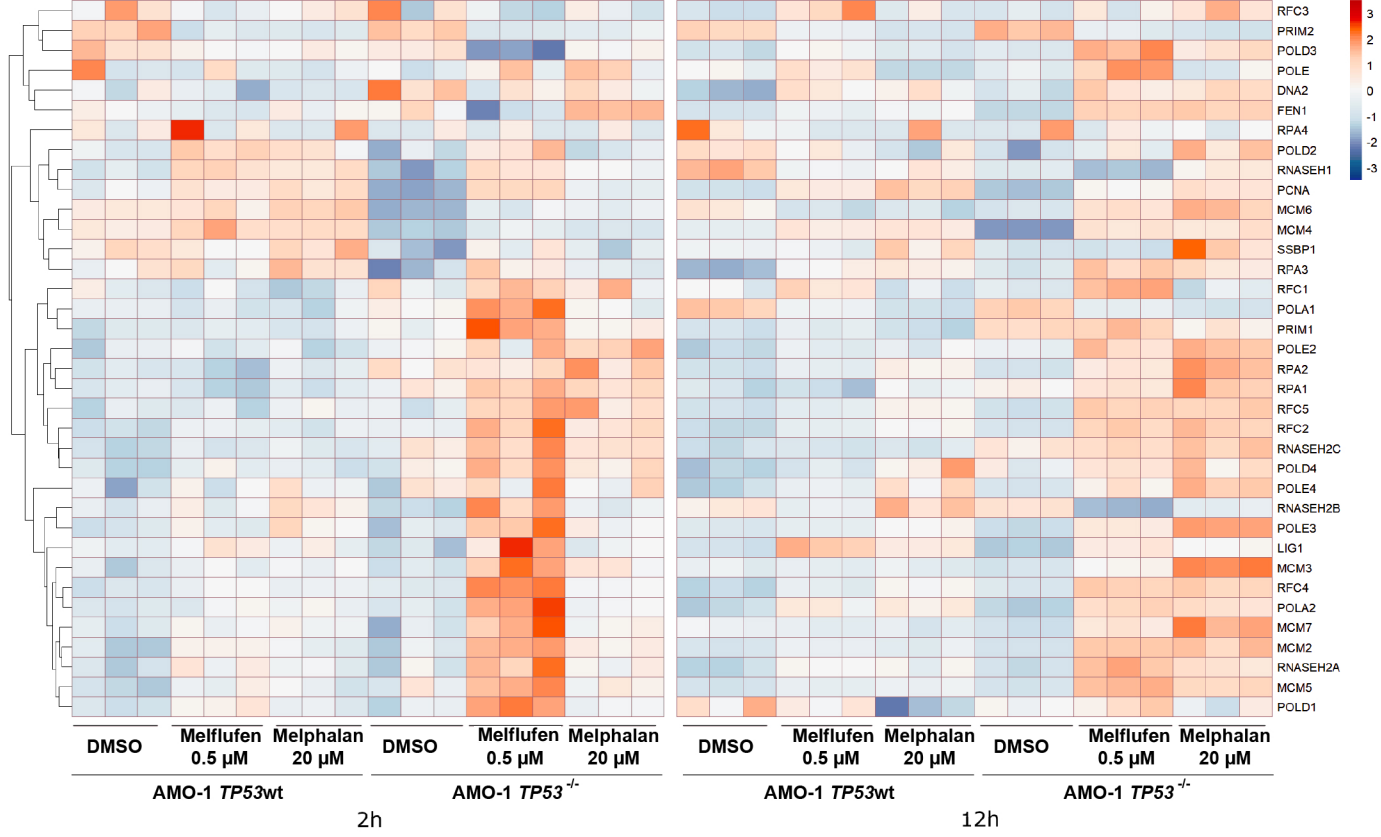

Nucleotide excision repair

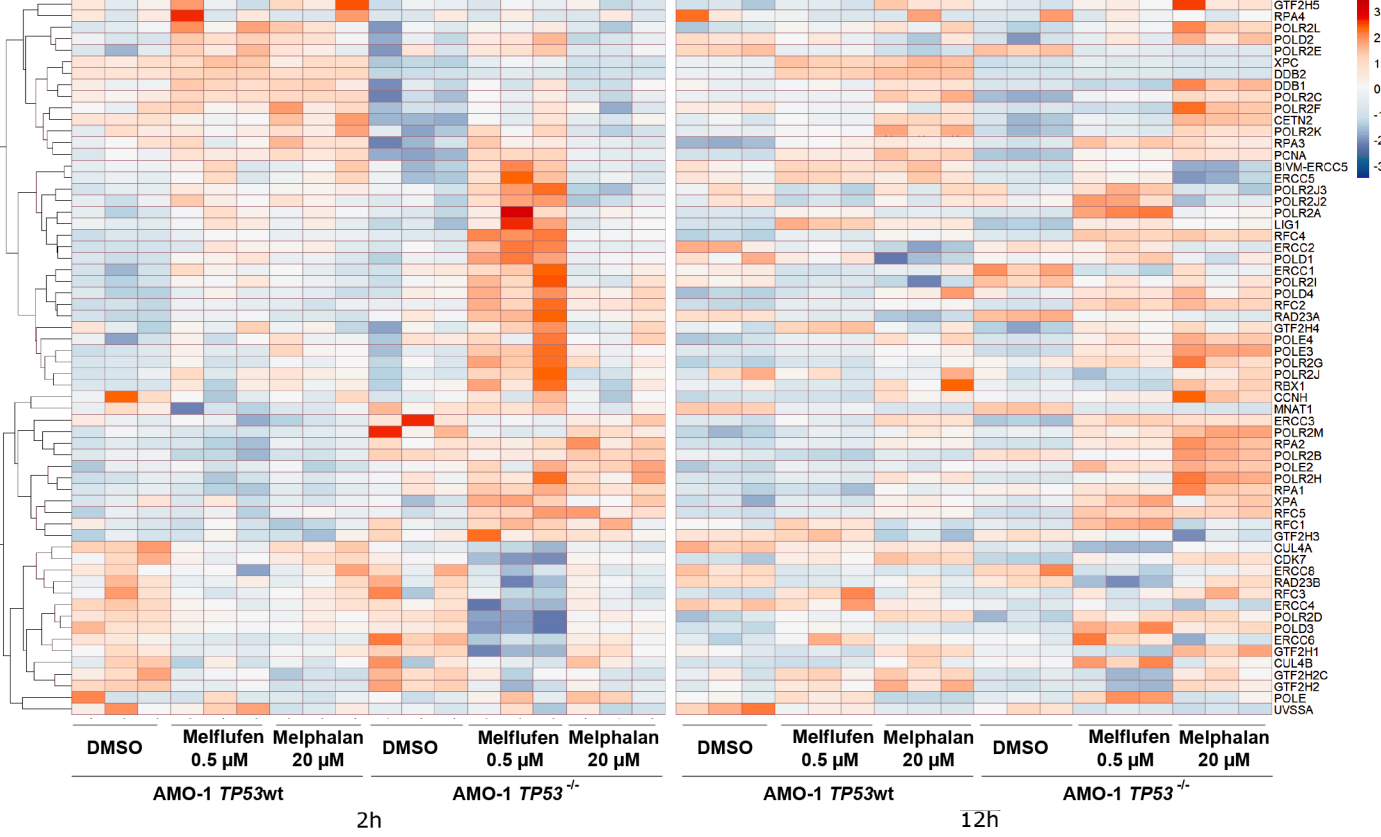

Base excision repair

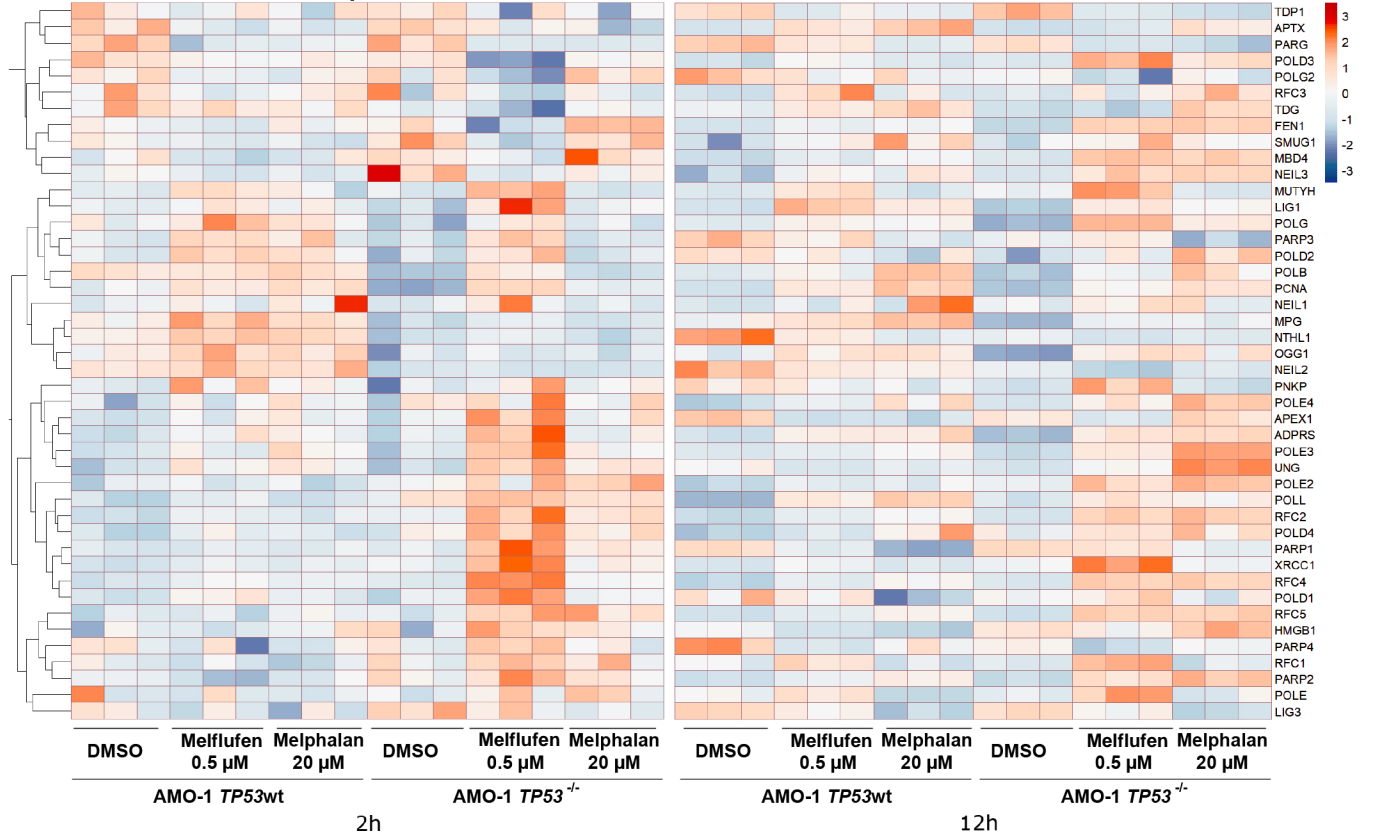

Supplement: Supplementary file 12 — Supplementary Material 12 [file 40164_2025_729_MOESM12_ESM.pdf]
