# Supplementary material for: Efficacy of melflufen in multiple myeloma with mutated or deleted TP53
Source: Exp Hematol Oncol. 2025 Dec 23;14:138. doi: 10.1186/s40164-025-00729-1 (PMC12729255; doi:10.1186/s40164-025-00729-1)
Supplement: Supplementary file 7 — Supplementary Material 7 [file 40164_2025_729_MOESM7_ESM.pdf]

**Table S4. Melflufen and melphalan EC50 and EC90 values in the AMO-1 isogenic *TP53* wt and *TP53* -/- cell lines**

| | Melflufen | | Melphalan | |
|---|---|---|---|---|
| Cell line | EC50, µM | EC90, µM | EC50, µM | EC90, µM |
| AMO-1 *TP53* wt | 0.1 | 0.5 | 9.0 | ≥20 |
| AMO-1 *TP53* -/- | 0.6 | 2.5 | ≥20 | ≥20 |

Cell lines were treated with either melflufen or melphalan at an 8-point drug concentration range to determine EC50 or EC90 values using a viability
