# Supplementary material for: Efficacy of melflufen in multiple myeloma with mutated or deleted TP53
Source: Exp Hematol Oncol. 2025 Dec 23;14:138. doi: 10.1186/s40164-025-00729-1 (PMC12729255; doi:10.1186/s40164-025-00729-1)
Supplement: Supplementary file 9 — Supplementary Material 9 [file 40164_2025_729_MOESM9_ESM.pdf]

Table S5. Patient baseline demographic and disease characteristics in the del(17p) patient subpopulation from the OCEAN trial

Baseline demographics

	Mel - del17p, N=33	Mel - Not del17p, N=213	Pom - del17p, N=37	Pom - Not del17p, N=212
**Age**	68 (45-85)	67 (41-91)	69 (43-83)	68 (39-87)
**Agegroup**				
**<65**	11 (33.3)	85 (39.9)	11 (29.7)	74 (34.9)
**65-74**	19 (57.6)	94 (44.1)	19 (51.4)	106 (50.0)
**75+**	3 (9.1)	34 (16.0)	7 (18.9)	32 (15.1)
**Male**	20 (61)	119 (56)	19 (51)	121 (57)
**Race**				
**WHITE**	30 (90.9)	194 (91.1)	32 (86.5)	190 (89.6)
**BLACK OR AFRICAN AMERICAN**	0 (0.0)	4 (1.9)	0 (0.0)	4 (1.9)
**ASIAN**	1 (3.0)	7 (3.3)	5 (13.5)	8 (3.8)
**NATIVE HAWAIIAN OR OTHER PACIFIC ISLANDER**	0 (0.0)	0 (0.0)	0 (0.0)	0 (0.0)
**OTHER**	0 (0.0)	1 (0.5)	0 (0.0)	0 (0.0)
**NOT REPORTED**	0 (0.0)	0 (0.0)	0 (0.0)	1 (0.5)
**UNKNOWN**	2 (6.1)	7 (3.3)	0 (0.0)	9 (4.2)

Disease characteristics

	Mel - del17p, N=33	Mel - Not del17p, N=213	Pom - del17p, N=37	Pom - Not del17p, N=212
**Years from diagnosis, median (range)**	4.5 (1.5-21.5)	3.9 (0.5-26.3)	3.8 (1.2-12.5)	3.9 (0.4-25.2)
**Prior treatment regimens, median (range)**	3 (2-4)	3 (2-4)	2 (2-4)	3 (2-4)
**Documented refractory status, n(%)**				
**Lenalidomide**	33 (100)	213 (100)	37 (100)	212 (100)
**Pomalidomide**	0 (0)	0 (0)	0 (0)	0 (0)
**Bortezomib**	15 (45)	96 (45)	22 (59)	103 (49)
**Carfilzomib**	9 (27)	38 (18)	9 (24)	28 (13)
**Daratumumab**	3 (9)	45 (21)	7 (19)	31 (15)
**Alkylator refractory, n(%)**	8 (24)	70 (33)	9 (24)	66 (31)
**Melphalan exposed**	11 (33)	50 (23)	17 (46)	47 (22)
**Melphalan refractory**	2 (6)	13 (6)	4 (11)	19 (9)
**Previous stem cell transplant, n(%)**	19 (58)	106 (50)	15 (41)	105 (50)
**International Staging System at Baseline, n(%)**				
**I**	10 (30)	109 (51)	14 (38)	110 (52)
**II**	19 (58)	75 (35)	18 (49)	76 (36)
**III**	4 (12)	29 (14)	5 (14)	26 (12)
**High-risk cytogenetics, n(%)**	32 (97)	51 (24)	33 (89)	53 (25)
**Extramedullary disease (EMD), n(%)**	2 (6)	28 (13)	4 (11)	22 (10)
**ECOG at baseline, n(%)**				
**0**	9 (27)	81 (38)	11 (30)	81 (38)
**1**	17 (52)	113 (53)	22 (59)	114 (54)
**2**	7 (21)	19 (9)	4 (11)	17 (8)
**TTP following ASCT**				
**No transplant**	14 (42)	107 (50)	22 (59)	107 (50)
**<1y**	4 (12)	27 (13)	6 (16)	26 (12)
**1-2y**	8 (24)	39 (18)	4 (11)	47 (22)
**2-3y**	4 (12)	19 (9)	1 (3)	17 (8)
**>3y**	3 (9)	21 (10)	4 (11)	15 (7)
