# Supplementary material for: Efficacy of melflufen in multiple myeloma with mutated or deleted TP53
Source: Exp Hematol Oncol. 2025 Dec 23;14:138. doi: 10.1186/s40164-025-00729-1 (PMC12729255; doi:10.1186/s40164-025-00729-1)
Supplement: Supplementary file 11 — Supplementary Material 11 [file 40164_2025_729_MOESM11_ESM.pdf]

Table S7. Patient baseline demographic and disease characteristics in the del(17p) and/or TP53 gene mutated patient subpopulations from the OCEAN trial.

Baseline demographics

	Melflufen - TP53 mutant, N=15	Melflufen - TP53 wild type , N=62	Pomalidomide - TP53 mutant, N=18	Pomalidomide - TP53 wild type, N=50
**Age**	66 (51-80)	65 (41-84)	68 (43-83)	68 (46-80)
**Agegroup**				
**<65**	6 (40.0)	28 (45.2)	6 (33.3)	14 (28.0)
**65-74**	8 (53.3)	27 (43.5)	9 (50.0)	27 (54.0)
**75+**	1 (6.7)	7 (11.3)	3 (16.7)	9 (18.0)
**Male**	6 (40)	32 (52)	9 (50)	25 (50)
**Race**				
**WHITE**	15 (100.0)	55 (88.7)	15 (83.3)	44 (88.0)
**BLACK OR AFRICAN AMERICAN**	0 (0.0)	0 (0.0)	1 (5.6)	1 (2.0)
**ASIAN**	0 (0.0)	4 (6.5)	2 (11.1)	3 (6.0)
**NATIVE HAWAIIAN OR OTHER PACIFIC ISLANDER**	0 (0.0)	0 (0.0)	0 (0.0)	0 (0.0)
**OTHER**	0 (0.0)	1 (1.6)	0 (0.0)	0 (0.0)
**NOT REPORTED**	0 (0.0)	0 (0.0)	0 (0.0)	0 (0.0)
**UNKNOWN**	0 (0.0)	2 (3.2)	0 (0.0)	2 (4.0)

Disease characteristics

	Melflufen - TP53 mutant, N=15	Melflufen - TP53 wild type , N=62	Pomalidomide - TP53 mutant, N=18	Pomalidomide - TP53 wild type, N=50
**Years from diagnosis, median (range)**	4.5 (1.5-8.4)	4.6 (0.5-17.8)	4.3 (1.2-12.5)	4.4 (0.4-13.7)
**Prior treatment regimens, median (range)**	3 (2-4)	3 (2-4)	3 (2-4)	3 (2-4)
**Documented refractory status, n(%)**				
**Lenalidomide**	15 (100)	62 (100)	18 (100)	50 (100)
**Pomalidomide**	0 (0)	0 (0)	0 (0)	0 (0)
**Bortezomib**	11 (73)	30 (48)	10 (56)	22 (44)
**Carfilzomib**	0 (0)	6 (10)	3 (17)	7 (14)
**Daratumumab**	1 (7)	14 (23)	2 (11)	7 (14)
**Alkylator refractory, n(%)**	6 (40)	25 (40)	6 (33)	8 (16)
**Melphalan exposed**	5 (33)	11 (18)	7 (39)	15 (30)
**Melphalan refractory**	2 (13)	5 (8)	2 (11)	3 (6)
**Previous stem cell transplant, n(%)**	9 (60)	33 (53)	10 (56)	29 (58)
**International Staging System at Baseline, n(%)**				
**I**	6 (40)	37 (60)	4 (22)	31 (62)
**II**	7 (47)	18 (29)	13 (72)	15 (30)
**III**	2 (13)	7 (11)	1 (6)	4 (8)
**High-risk cytogenetics, n(%)**	13 (87)	16 (26)	14 (78)	18 (36)
**Extramedullary disease (EMD), n(%)**	1 (7)	14 (23)	2 (11)	4 (8)
**ECOG at baseline, n(%)**				
**0**	6 (40)	21 (34)	4 (22)	20 (40)
**1**	6 (40)	32 (52)	12 (67)	26 (52)
**2**	3 (20)	9 (15)	2 (11)	4 (8)
**TTP following ASCT**				
**No transplant**	6 (40)	29 (47)	8 (44)	21 (42)
**<1y**	3 (20)	4 (6)	4 (22)	6 (12)
**1-2y**	4 (27)	15 (24)	3 (17)	19 (38)
**2-3y**	1 (7)	7 (11)	2 (11)	3 (6)
**>3y**	1 (7)	7 (11)	1 (6)	1 (2)
