# Supplementary material for: Efficacy of melflufen in multiple myeloma with mutated or deleted TP53
Source: Exp Hematol Oncol. 2025 Dec 23;14:138. doi: 10.1186/s40164-025-00729-1 (PMC12729255; doi:10.1186/s40164-025-00729-1)
Supplement: Supplementary file 13 — Supplementary Material 13 [file 40164_2025_729_MOESM13_ESM.pdf]

A

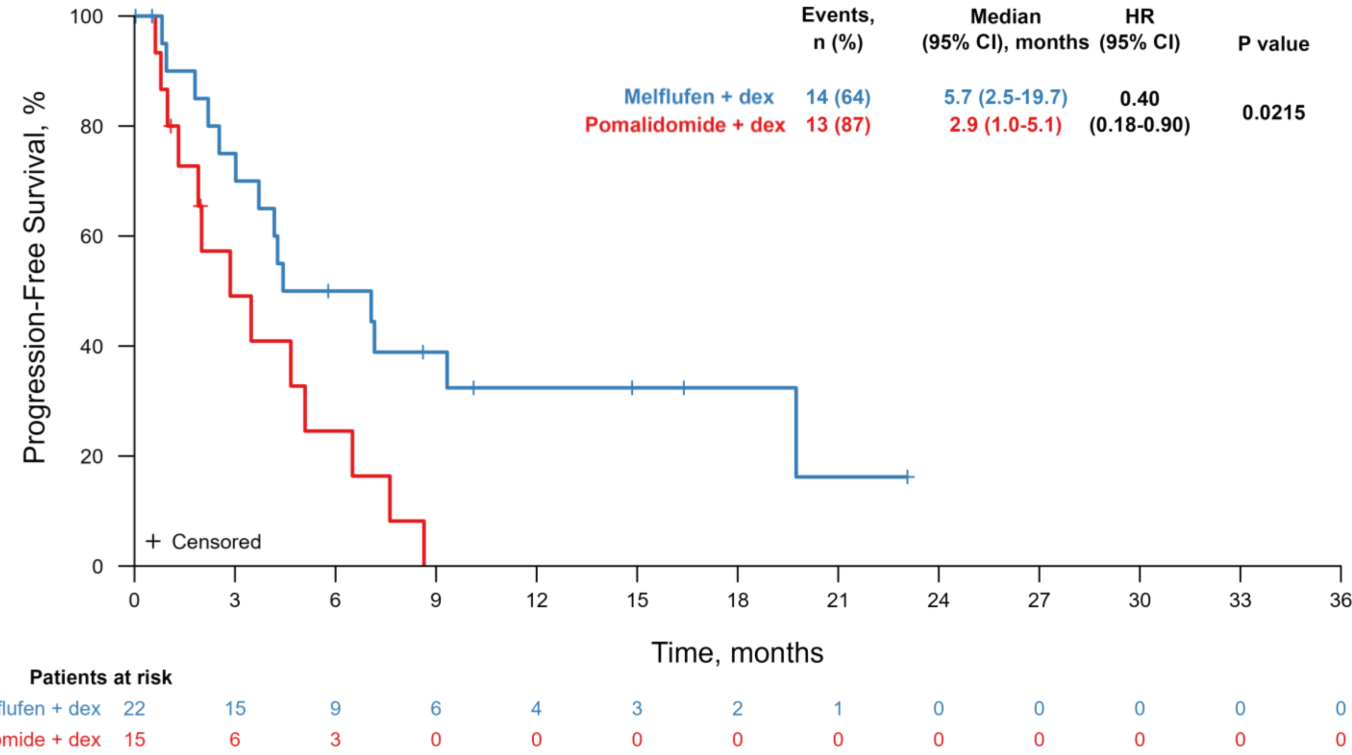

B

	Melflufen N=22	Pomalidomide N=15
**Best Overall Confirmed Response, n (%)**		
sCR	0 (0.0)	0 (0.0)
CR	0 (0.0)	0 (0.0)
VGPR	3 (13.6)	0 (0.0)
PR	2 (9.1)	0 (0.0)
MR	6 (27.3)	1 (6.7)
SD	4 (18.2)	7 (46.7)
PD	3 (13.6)	5 (33.3)
NE	4 (18.2)	2 (13.3)
**Overall Confirmed Response Rate**		
sCR+CR+VGPR+PR, n (%)	5 (22.7)	0 (0.0)
[95% CI]	7.8-45.4	0.0-21.8
**Unstratified p-value**	0.0502	
