# Supplementary material for: Efficacy of melflufen in multiple myeloma with mutated or deleted TP53
Source: Exp Hematol Oncol. 2025 Dec 23;14:138. doi: 10.1186/s40164-025-00729-1 (PMC12729255; doi:10.1186/s40164-025-00729-1)
Supplement: Supplementary file 14 — Supplementary Material 14 [file 40164_2025_729_MOESM14_ESM.pdf]

A

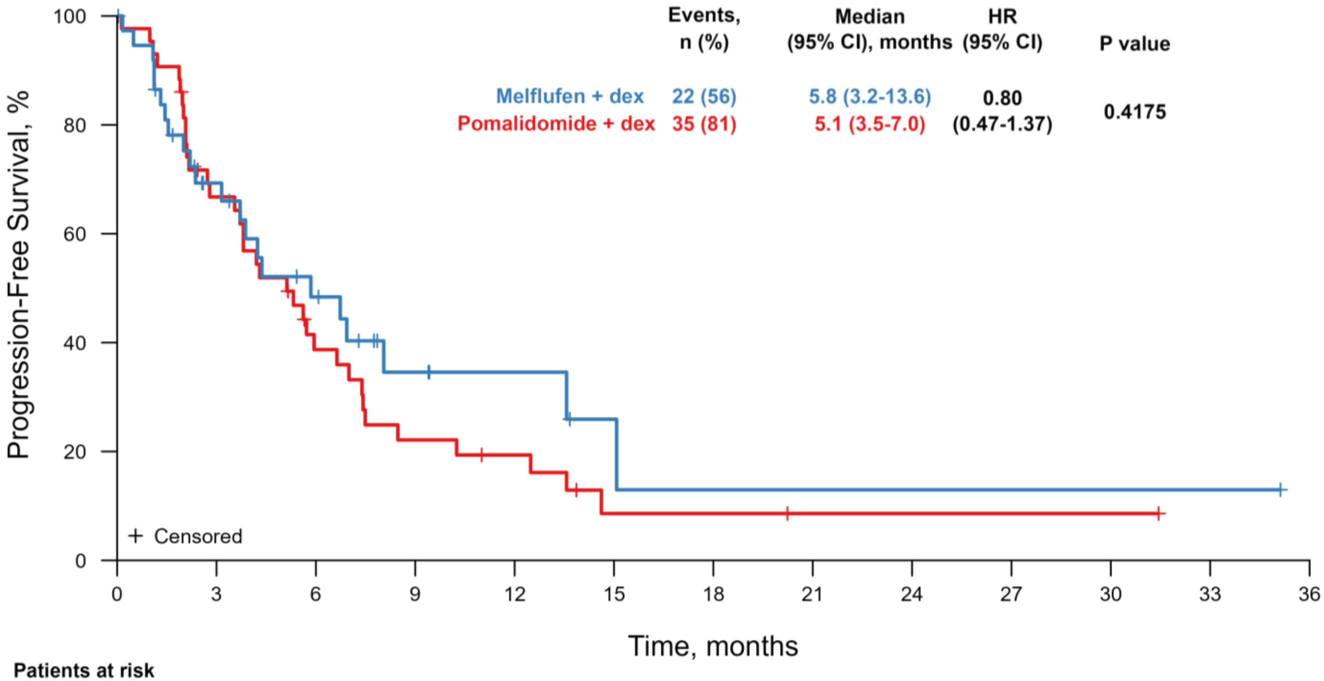

Patients at risk

Melflufen + dex	39	21	13	6	4	2	1	1	1	1	1	1	0
Pomalidomide + dex	43	27	14	8	6	2	2	1	1	1	1	0	0

B

	Melflufen N=39	Pomalidomide N=43
**Best Overall Confirmed Response, n (%)**		
sCR	0 (0.0)	0 (0.0)
CR	1 (2.6)	0 (0.0)
VGPR	5 (12.8)	1 (2.3)
PR	4 (10.3)	9 (20.9)
MR	6 (15.4)	11 (25.6)
SD	9 (23.1)	11 (25.6)
PD	11 (28.2)	11 (25.6)
NE	3 (7.7)	0 (0.0)
**Overall Confirmed Response Rate**		
sCR+CR+VGPR+PR, n (%)	10 (25.6)	10 (23.3)
[95% CI]	13.0-42.1	11.8-38.6
**Unstratified p-value**	0.8029	
