# Supplementary material for: Efficacy of melflufen in multiple myeloma with mutated or deleted TP53
Source: Exp Hematol Oncol. 2025 Dec 23;14:138. doi: 10.1186/s40164-025-00729-1 (PMC12729255; doi:10.1186/s40164-025-00729-1)
Supplement: Supplementary file 15 — Supplementary Material 15 [file 40164_2025_729_MOESM15_ESM.docx]

**SUPPLEMENTARY MATERIALS FOR:**

**Efficacy of melflufen in multiple myeloma with mutated or deleted *TP53***

**SUPPLEMENTARY MATERIALS AND METHODS**

**Patient samples**

Bone marrow (BM) aspirates (n=24) from the Finnish Hematology Registry and Biobank were collected from 23 multiple myeloma (MM) patients after informed consent using protocols approved by an ethical committee and in accordance with the Declaration of Helsinki. Patients received median 2 (range 1-5) lines of therapy prior sampling. The study was approved by an ethical committee of the Helsinki University Hospital (permit numbers 239/13/03/00/2010, 303/13/03/01/2011). BM mononuclear cells (BM-MNCs) were purified by Ficoll gradient centrifugation and cryopreserved prior to analyses.

**Ex vivo drug sensitivity testing with patient cells by flow cytometry**

For flow cytometry-based drug sensitivity testing (FC-DSRT), viably frozen MM patient BM-MNC were thawed, freezing solution washed away, cell pellets suspended in conditioned medium (RPMI 1640 medium supplemented with 10% FBS, 2 mM L-glutamine, penicillin (100 U/ml), streptomycin (100 μg/ml) and 25% conditioned medium from the HS-5 human bone marrow stromal cell line),^1^ DNase I (Promega) treated for 60 min, and cultured in conditioned medium overnight. Cells were filtered through a 70 μm cell strainer (Fisher Scientific), cell viability measured, and cells plated in parallel on pre-drugged 96-well plates (100,000 viable cells/well in 100 μl) with following drug concentration ranges: melflufen and melphalan (0.1-100,000 nM), cyclophosphamide (0.1-10,000 nM) in duplicates. If cell viability was poor (<50%), the Dead Cell Removal kit (Miltenyi Biotec) was used prior to plating the cells. Stock solutions (10,000 μM) of melflufen (Oncopeptides AB) and melphalan (Sigma-Aldrich), that had been prepared in advance and stored at -80 °C, were thawed and diluted in conditioned medium to desired concentrations shortly before the drug testing to minimize auto-hydrolysis of melflufen and melphalan (exchange of Cl with OH at the *N*-mustard part of the molecules). Melflufen and melphalan were added to the 96-well plates by manually pipetting only after the cells had been plated, just prior to the start of the drug sensitivity test. The cells were incubated with the drugs for 72 h at 37 °C and 5% CO_2_. Following 72 h incubation with the drugs, cells were centrifuged (500xg, 6 min) in the 96-well plates and media discarded by inverting the plate. Cells were suspended in 25 μl of antibody mix containing staining buffer (5% FBS in Dulbecco's phosphate-buffered saline) and the following two antibodies (BD Biosciences): CD138 (BV605, clone MI15, dilution 1:100) and CD38 (BV786, clone HIT2, 1:100). Cells were stained for 30 min at room temperature in the dark and subsequently washed with 100 μl staining buffer followed by centrifugation (500xg, 6 min) and supernatant removal by inverting the plate. Apoptotic and dead cells were discriminated by 7-aminoactinomycin D (7-AAD) and PE-Annexin V (BD Biosciences) staining with both dyes diluted 1:50 in 25 μl annexin V binding buffer. The plates were incubated for 10 min at room temperature before FC analysis. FC analysis was performed using the IntelliCyt iQue Screener PLUS instrument (Sartorius). MM patient sample BM-MNCs were acquired for analysis from each 96-well plate well by the iQue Screener PLUS instrument using a 17 s sip time per well and a pump speed of 32 revolutions per minute. ForeCyt software (Sartorius) was used to gate cells and acquire population counts. Analysis was done from viable singlet cells. The cell count of a well was normalized to its 6 adjacent DMSO controls, from which the highest and lowest value were excluded and normalized values were used for calculating cell viability percentages for each tested drug concentration, and dose response curves drawn. Drug sensitivity was quantified using the drug sensitivity score (DSS), a normalized metric that integrates multiple dose–response parameters (such as IC50, maximum response, and curve slope parameters) into a single value. DSS was calculated from fitted dose–response curves using the area under it, providing a robust measure of overall drug effect across tested concentrations.^2^ For analyses, the samples were divided into different melflufen sensitivity groups based on the melflufen DSS values of the samples: high (HS, DSS>40; n=8), intermediate (IS, 40<DSS<31; n=8), and low (LS, DSS>31; n=8).

**Single-cell gene expression analysis**

For single cell RNA sequence (scRNAseq) analysis, the MM patient BM-MNCs were sorted based on cell viability (7-AAD, BD Biosciences) and CD138+ surface expression (APC, clone MI15, BD Biosciences) using fluorescence-activated cell sorting (BD Influx Cell Sorter, BD Biosciences). CD138+ and CD138− sorted cell fractions were mixed at a 1:1 ratio with a maximum of 800,000 cells per sample. If there were fewer than 400,000 CD138+ cells, then all CD138+ cells were mixed with 400,000 CD138- cells. scRNAseq library preparation was performed on the mixed CD138+ and CD138+ cell samples using the Chromium Single Cell 3′ Gene Expression v3 reagent kit (10x Genomics). The gel beads in emulsion (GEM) generation, cDNA amplification and library preparation were performed according to the manufacturer’s instructions. The prepared libraries were then sequenced on the Illumina NovaSeq 6000 instrument (Illumina) with paired-end reads 28 bp and 89 bp.

Analysis of scRNA-seq data was performed in R Studio environment, using Seurat package (version 4.0.1).^3^ Upon data uploading, quality control of cells was done. We used percentage of mitochondrial genes (< triple median or 30%, whichever is less), number of unique genes (>500), number of transcripts (< triple median), and log10 transformed ratio of number of transcripts to number of unique genes (>0.8) to remove dead and late apoptotic cells, as well as multiplets from the subsequent analysis. Samples were integrated, using anchoring procedure, normalized and variance stabilized, and clustered, based on construction of a Shared Nearest Neighbor Graph, which utilized a dataset with dimensions reduced by the prior principal component analysis. Clusters were identified, based on the gene expression patterns, and divided into plasma cells (CD138+ pre-sorted fraction) and cells of other types (CD138- pre-sorted fraction) for further analysis. After division of the initial dataset, the new datasets were re-integrated, normalized, and clustered. Additionally, for the plasma cell dataset, we filtered out immunoglobulin genes. Final clustering results were checked for the distribution of cells, based on cell cycle phase and mitochondrial content, and annotated, using the genes expressed differently between the given and all the other clusters.

For the analysis of differentially expressed genes (DGE), we sequentially compared HS *vs.* IS, IS *vs.* LS, and HS *vs.* LS samples, filtering out non-monotonous changes in gene expression between those groups, and using a threshold of 0.2 for log2-transfored fold change values. The identification of DGE was done by the means of MAST framework. ^4^

For gene set enrichment analysis, clusterProfiler (v.3.18.1)^5^ and ReactomePA (v. 1.34.0)^6^ packages were used. We utilized Reactome^7^, KEGG^8^ and MSigDB^9,10^ datasets. Differentially expressed genes from melflufen comparison, with preliminary subtracted genes from DGE analysis for melphalan, and retained genes altered due to the disease stage, were used as an input to cluster genes, based on their belonging to the gene sets, respectively to their functions, regulation, or expression patterns. Data link NCBI GEO (http://www.ncbi.nlm.nih.gov/geo) accession number GSE263201.

**Cell lines, cell culture and cytotoxicity**

The AMO-1 cell line was obtained from DSMZ (Braunschweig, Germany). Generation and characterization of *TP53* deficient AMO-1 clones is described in Munawar et al., 2019.^11^ Standard cell culture was performed in RPMI-1640 medium supplemented with 10% fetal bovine serum (FBS), 1 mM sodium pyruvate, 2 mM glutamine, 100 U/ml penicillin, and 100 μg/ml streptomycin. Cells were kept at 5% CO_2_ and 37 °C. Cells were cultured on 96-well plates in 200 µl culture medium at 2 M/ml concentration for 72 h exposure period before measuring the number of viable cells. Cell viability was determined using the CellTiter-Glo® 2.0 assay (Promega), AlamarBlue^TM^ reagent (Invitrogen) or cell death assays (annexin V/propidium iodide) according to the manufacturers’ protocols. Drug efficacy was determined by EC50 parameter for each cell line. Difference in EC50 values between parental *TP53*wt and *TP53*^-/-^ cell lines was denoted as ΔEC50, where ΔEC50 > 0 means higher resistance to a drug.

**Compounds and stock solutions**

Melflufen (Oncopeptides AB) was freshly prepared in DMSO before application. Melphalan (Sigma-Aldrich, M2011) stock solution was prepared in acidified ethanol. Cyclophosphamide (N,N-bis(2-chloroethyl)-4-hydroperoxy-2-oxo-1,3,25-oxazaphosphinan-2-amine,TRC (product number TRC-H714675) was freshly prepared in DMSO.

**RNA preparation and RNA-seq analysis**

AMO1 *TP53* wild type cells or the *TP53^-/-^* knock out clonal subline AMO-1 TP53^-/-^ were treated with melphalan or melflufen at different concentrations and timepoints. RNA was isolated according to the RNeasy plus protocol, quantified with Nanodrop (Thermo Scientific), and quality assessed with the Agilent 2100 Bioanalyzer. DNA libraries were prepared using the TruSeq Stranded mRNA Library Preparation Kit (Illumina) and sequencing performed on the NextSeq 500 platform (Illumina). Base calling was performed by Real-Time Analysis (RTA) version 2.4.11 or 2.11.3 and demultiplexed FASTQ files were generated with bcl2fastq2 v2.20.0.422 (Illumina). To assure high sequence quality, Illumina reads were quality- and adapter-trimmed via Cutadapt^12,13^ version 2.5 using a cutoff Phred score of 20 in NextSeq mode and reads without any remaining bases were discarded (command line parameters: --nextseq-trim=20 -m 1 -a AGATCGGAAGAGCACACGTCTGAACTCCAGTCAC). Processed reads were subsequently mapped to the human genome (GRCh38.p14) using STAR v2.7.2b with default parameters based on RefSeq annotation version 110 for GRCh38.p14.^14^ Read counts on exon level summarized for each gene were generated using featureCounts v1.6.4 from the Subread package.^15^ Multi-mapping and multi-overlapping reads were counted strand-specific and reversely stranded with a fractional count for each alignment and overlapping feature (command line parameters: -s 2 -t exon -M -O --fraction). The count output was utilized to identify differentially expressed genes using DESeq2^15^ version 1.24.0. Read counts were normalized by DESeq2 and fold-change shrinkage was applied by setting the parameter “betaPrior=TRUE”. Differential expression of genes was assumed at an adjusted p-value (padj) after Benjamini-Hochberg correction < 0.05 and |log2FoldChange| ≥ 0.5.

**DNA damage signaling and cell apoptosis analyses**

AMO-1 *TP53* wild type and AMO-1 *TP53*^-/-^ cells were treated with melflufen, melphalan, or cyclophosphamide at the indicated concentrations and time points. H2AX phosphorylation at Ser139 (γ-H2AX) for DNA damage signaling was analyzed by flow cytometry. The following protocol was applied for γ-H2AX staining: Cells were fixed in Flow Cytometry Fixation Buffer (R&D Systems) and stored in 70% ethanol at -20°C overnight. Prior to incubation with Alexa Fluor 488-conjugated phosphorylated H2AX antibody (Sigma-Aldrich) or Mouse IgG1 (R&D Systems), cells were blocked with Human Seroblock (Bio-Rad Laboratories).

For apoptosis analysis, cells were stained for annexin V-APC and PI (Invitrogen) as follows; cells were suspended in staining mix containing Annexin V APC (Invitrogen) and PI (Invitrogen), fixed in Flow Cytometry Fixation Buffer and resuspended in Annexin V binding buffer (Invitrogen).

All samples were analyzed with the FACSCanto II flow cytometer (BD Biosciences). Data were analyzed using FlowJo software (BD Biosciences).

**JC-1 mitochondria membrane potential assay**

AMO-1 *TP53* wild type and AMO-1 *TP53*^-/-^ cells at 1x10^5^/mL were treated with melflufen, melphalan or cyclophosphamide at the indicated concentrations and cells were harvested after 2h or 4h. The JC-1 mitochondrial membrane potential assay was performed according to the manufacturer’s protocol (MitoProbe™ JC-1 Assay Kit for Flow Cytometry (M34152), Molecular Probes, Eugene, OR). Following drug treatment, the cells were stained with JC-1 (5,5’,6,6’-tetrachloro-1,1’,3,3’-tetra-ethyl-benzimidazolyl-carbocyanine iodide) reagent for 15 min at 37 °C, pelleted by centrifugation (4 min/250xg), washed with PBS and resuspended in PBS. The ratio of green to red fluorescence was determined with a Spectra Max i3X (Molecular Devices) plate reader. Data was generated with 488 nm excitation and using 529 nm and 590 nm emission filters. Obtained measurement data was analyzed to calculate the mitochondria membrane potential changes upon drug treatment.

**Statistical analyses of the OCEAN trial data**

Primary analyses of tumor response and progression-dependent endpoints of patients enrolled in the OCEAN trial (NCT03151811) were based on response assessments by an Independent Review Committee. All tumor response and progression-dependent endpoints were assessed using the IMWG Uniform Response Criteria (IMWG-URC). PFS was defined as time (months) from date of randomization to either confirmed disease progression or death due to any cause. The overall response rate (ORR) was defined as the proportion of patients for whom the best overall confirmed response was stringent complete response (sCR), complete response (CR), very good partial response (VGPR), or partial response (PR). Post hoc analyses were performed to examine associations between progression-free survival (PFS) and chromosome 17 deletion status and/or *TP53* gene mutation status. Furthermore, another post-hoc analysis was carried out to study the patient responder status in the chromosome 17 deletion population.

**Assessment of *TP53* mutation status of OCEAN trial patients**

*TP53* mutation status of OCEAN trial patients was determined by next generation sequencing (NGS) using Almac Genomic Services. In brief, Almac´s NGS v2 TP53 Assay has been performed what detects germline and somatic single nucleotide variants (SNV), small insertion and small deletion events within the promoter region, the coding and non-coding regions of exons one to ten, a portion of exon eleven, and the intron-exon boundaries of TP53 isoform a (NM_000546) using the Illumina MiSeq platform. Sample preparation for the sequencing has been performed as followed; from patients, who consented, bone marrow aspirates CD138+ purified plasma cells had been frozen shipped to Almac Genomic Services. From CD138+ cells, DNA was extracted using QIAamp® DNA Blood Midi Kit (QIAGEN©), DNA quality was checked according to Almac`s Qubit quality control. NGS v2 TP53 assay was performed using the TruSeq Custom Amplicon Kit Dx and custom TP53 oligo panel. Analysis was performed as follows: sequencing, demultiplexing, FASTQ file generation, sequence alignment and variant calling was automatically performed onboard the MiSeq system. Sequencing run, sample and control QC was performed using the Almac Genomic Services developed software, named Almac TP53 CTA v2 Analysis. All detected SNVs were classified and aligned with the National Library of Medicine, NIH database, for ClinVar genomic variation database and clinical relevance to classify mutation type into benign or pathogenic. The *TP53* mutation status of the patient samples used for the *ex vivo* drug sensitivity testing was determined using exome sequencing of DNA from CD138+ cells as previously described.^16^

**Assessment of del(17p) status**

The del(17p) status had been determined from bone marrow at baseline level (entry) of the clinical study by interphase Fluorescence In-Situ Hybridization (iFISH) in accordance with Good Clinical Practice (GCP). No cutoff was applied and all patients with del(17p) cytogenetics were included in the analyses. The presence of del(17p) in plasma cells varied between 8-90%. The del(17p) status of the patient samples used for the *ex vivo* drug sensitivity testing was determined using iFISH as previously described.^16^

Parts of the manuscript text were edited for clarity and grammar using Copilot, a large language model developed by Microsoft (accessed July 2025). All content was reviewed and approved by the authors.

**SUPPLEMENTARY REFERENCES**

1. Roecklein BA, Torok-Storb B. Functionally distinct human marrow stromal cell lines immortalized by transduction with the human papilloma virus E6/E7 genes. *Blood*. 1995;85(4):997-1005.

2. Yadav B, Pemovska T, Szwajda A, et al. Quantitative scoring of differential drug sensitivity for individually optimized anticancer therapies. *Scientific reports*. 2014;4:5193.

3. Hao Y, Hao S, Andersen-Nissen E, et al. Integrated analysis of multimodal single-cell data. *Cell*. 2021;184(13):3573-3587 e3529.

4. Finak G, McDavid A, Yajima M, et al. MAST: a flexible statistical framework for assessing transcriptional changes and characterizing heterogeneity in single-cell RNA sequencing data. *Genome Biol*. 2015;16:278.

5. Yu G, Wang LG, Han Y, He QY. clusterProfiler: an R package for comparing biological themes among gene clusters. *OMICS*. 2012;16(5):284-287.

6. Yu G, He QY. ReactomePA: an R/Bioconductor package for reactome pathway analysis and visualization. *Mol Biosyst*. 2016;12(2):477-479.

7. Jassal B, Matthews L, Viteri G, et al. The reactome pathway knowledgebase. *Nucleic Acids Res*. 2020;48(D1):D498-D503.

8. Kanehisa M, Goto S. KEGG: kyoto encyclopedia of genes and genomes. *Nucleic Acids Res*. 2000;28(1):27-30.

9. Liberzon A, Subramanian A, Pinchback R, Thorvaldsdottir H, Tamayo P, Mesirov JP. Molecular signatures database (MSigDB) 3.0. *Bioinformatics*. 2011;27(12):1739-1740.

10. Subramanian A, Tamayo P, Mootha VK, et al. Gene set enrichment analysis: a knowledge-based approach for interpreting genome-wide expression profiles. *Proc Natl Acad Sci U S A*. 2005;102(43):15545-15550.

11. Munawar U, Roth M, Barrio S, et al. Assessment of TP53 lesions for p53 system functionality and drug resistance in multiple myeloma using an isogenic cell line model. *Sci Rep*. 2019;9(1):18062.

12. Dobin A, Davis CA, Schlesinger F, et al. STAR: ultrafast universal RNA-seq aligner. *Bioinformatics*. 2013;29(1):15-21.

13. Martin M. Cutadapt removes adapter sequences from high-throughput sequencing reads. 2011;17(3):10-12.

14. Liao Y, Smyth GK, Shi W. featureCounts: an efficient general purpose program for assigning sequence reads to genomic features. *Bioinformatics*. 2014;30(7):923-930.

15. Love MI, Huber W, Anders S. Moderated estimation of fold change and dispersion for RNA-seq data with DESeq2. *Genome Biol*. 2014;15(12):550.

16. Majumder MM, Silvennoinen R, Anttila P, et al. Identification of precision treatment strategies for relapsed/refractory multiple myeloma by functional drug sensitivity testing. Oncotarget 2017;8:56338-56350.
